# Superinduction of immunosuppressive glioblastoma extracellular vesicles by IFN-γ through PD-L1 and IDO1

**DOI:** 10.1093/noajnl/vdac017

**Published:** 2022-02-15

**Authors:** Mi-Yeon Jung, Abudumijiti Aibaidula, Desmond A Brown, Benjamin T Himes, Luz M Cumba Garcia, Ian F Parney

**Affiliations:** Department of Neurological Surgery, Mayo Clinic College of Medicine, Rochester, Minnesota, USA; Department of Molecular Pharmacology and Experimental Therapeutics, Mayo Clinic College of Medicine, Rochester, MN, USA; Department of Neurological Surgery, Mayo Clinic College of Medicine, Rochester, Minnesota, USA; Department of Neurological Surgery, Mayo Clinic College of Medicine, Rochester, Minnesota, USA; Department of Immunology, Mayo Clinic College of Medicine, Rochester, Minnesota, USA; Department of Neurological Surgery, Mayo Clinic College of Medicine, Rochester, Minnesota, USA; Department of Immunology, Mayo Clinic College of Medicine, Rochester, Minnesota, USA; Department of Neurological Surgery, Mayo Clinic College of Medicine, Rochester, Minnesota, USA; Department of Immunology, Mayo Clinic College of Medicine, Rochester, Minnesota, USA

**Keywords:** extracellular vesicles (EVs), glioblastoma, IDO1, interferon-gamma (IFN-γ), PD-L1

## Abstract

**Background:**

Glioblastoma (GBM), the most common primary brain tumor, has a median survival of 15–16 months. Immunotherapy is promising but GBM-mediated immunosuppression remains a barrier. GBMs express the interferon-gamma (IFN-γ)-responsive immunosuppressive molecules programmed cell death ligand 1 (PD-L1) and indoleamine 2,3-dioxygenase 1 (IDO1). Extracellular vesicles (EVs) have also been implicated in GBM-mediated immunosuppression, in part through PD-L1. We therefore sought to determine if GBM IFN-γ exposure increased GBM EV-mediated immunosuppression and mechanisms underlying this.

**Methods:**

Human GBM-derived cells were cultured in the presence/absence of IFN-γ. EVs were harvested. PD-L1, IDO1, and EV-associated protein expression was assessed. GBM EVs (+/−IFN-γ) were cultured with healthy donor monocytes. Immunosuppressive myeloid-derived suppressor cell (MDSC) and nonclassical monocyte (NCM) frequency was determined. Impact of GBM (+/−IFN-γ) EV-treated monocytes on CD3/CD28-mediated T cell proliferation was assessed. The impact of PD-L1 and IDO1 knockdown in GBM EVs in this system was evaluated.

**Results:**

IFN-γ exposure increased PD-L1 and IDO1 expression in GBM cells and EVs without altering EV size or frequency. IFN-γ-exposed GBM EVs induced more MDSC and NCM differentiation in monocytes and these monocytes caused more T cell inhibition than IFN-γ-naive GBM EVs. PD-L1 and/or IDO1 knockdown in GBM cells abrogated the immunosuppressive effects of IFN-γ-exposed GBM EVs on monocytes.

**Conclusions:**

IFN-γ exposure such as might occur during an antitumor immune response results in superinduction of GBM EVs’ baseline immunosuppressive effects on monocytes. These effects are mediated by increased PD-L1 and IDO1 expression in GBM EVs. These data highlight mechanisms of GBM EV-mediated immunosuppression and identify therapeutic targets (PD-L1, IDO1) to reverse these effects.

**Key Points**

- IFN-γ increases GBM EV-mediated immunosuppressive monocyte (MDSC, NCM) induction.
- This is mediated by increased PD-L1 and IDO1 expression in IFN-γ-exposed GBM EVs.

**Importance of the Study**

Previous studies have demonstrated the expression of the IFN-γ-responsive immunosuppressive molecules PD-L1 and IDO1 by GBM cells. We have also shown that GBM EVs induce immunosuppressive myeloid cells (MDSCs, NCMs). In the present study, we demonstrate for the first time that GBM cells exposed to IFN-γ release EVs that are more immunosuppressive than EVs from IFN-γ-naive GBMs. This results in superinduction of MDSCs and NCMs with resulting increased T cell inhibition. Furthermore, these effects are dependent upon PD-L1 and IDO1 upregulation. IFN-γ expression is characteristic of activated antitumor immune responses. Therefore, IFN-γ-mediated superinduction of immunosuppressive GBM EVs may be a barrier to immunotherapy in this population. Importantly, both PD-L1 and IDO1 represent pharmacological targets for reversing GBM EV-mediated immunosuppression.

Glioblastoma (GBM) is the most common and aggressive primary central nervous system malignancy.^1^ Median survival is 15–16 months despite surgery, radiation, and chemotherapy.^2^ Ultimately, the disease is universally fatal with only 3%–5% of patients surviving to 5 years.^3^ Developing new treatment regimens has been slow and novel approaches such as the antiangiogenic drug bevacizumab^4^ and tumor treating fields^5^ have had only moderate success.

Cancer immunotherapy with checkpoint inhibitors has been FDA approved for multiple systemic malignancies.^6–9^ However, GBM immunotherapy has been disappointing. This partly reflects profound GBM-mediated immunosuppression.^10^ Understanding mechanisms causing GBM-mediated immunosuppression is critical to guide novel immunotherapeutic development.^7,11–13^

T cell regulation is a homeostatic balance between immune surveillance and self-tolerance while maintaining immunological memory.^14^ These regulatory processes are often co-opted in cancer leading to tumor-mediated immunosuppression.^15^ Immune checkpoint regulators play an important role in this. Programmed death protein-1 (PD-1)—a transmembrane protein expressed on the surface of leukocytes like T and B lymphocytes—has emerged as a critical immune checkpoint in cancer.^15–17^ PD-1 on cancer-specific cytotoxic lymphocytes binds to programmed cell death ligand 1 (PD-L1) expressed by cancer cells, initiating T cell apoptosis and functional exhaustion.^18^ A recent histopathological analysis of 235 gliomas found PD-1 expression in 31.5% of tumor-infiltrating lymphocytes and PD-L1 expression in 6.1% of tumors. Expression of both markers was significantly more frequent in higher grade tumors.^19^ GBM immune suppression is associated reduced circulating CD4 T cells and increased regulatory T cells (Tregs).^20^ PD-L1 expression by GBM cells is inducible by interferon-gamma (IFN-γ) exposure such as might occur during an antitumor immune response, therefore representing a pathway for immunosuppressive escape.^21^

Indoleamine 2,3-dioxygenase 1 (IDO1) is also implicated in T cell regulation in normal and pathologic states. IDO1 is an IFN-γ-inducible immune regulatory enzyme that catalyzes the catabolic conversion of tryptophan into kynurenine—a metabolic precursor of NAD^+^ and ATP.^22^ Upregulation of IDO1 in cancer results in tryptophan depletion and kynurenine accumulation which, in turn, induces T cell dysfunction and apoptosis.^22^ IFN-γ released in the tumor microenvironment in other cancers like melanoma results in increased expression of both IDO1 and PD-L1 and inhibition of antitumor immunity in a Treg-dependent manner.^23^ In hepatocellular carcinoma, tumor-derived IFN-γ leads to upregulation of PD-L1 and IDO1 and correlates with poor survival.^24^ While IDO1 and PD-L1 expression appear to overlap and are both regulated by IFN-γ, the precise relationship between these 2 regulators of T cell function remains unknown.

Finally, tumor-derived extracellular vesicles (EVs) are also implicated in GBM-mediated immunosuppression. EVs are nanosized lipid bilayer-encapsulated vesicles ranging from 30 to 1000 nm diameter. There is some controversy regarding nomenclature and sizes of the different types of vesicles, though small EVs (<100 nm) are often termed exosomes and larger EVs (>100–1000 nm) are often called microvesicles.^25^ Regardless, EVs have a critical role in cell-to-cell communication by facilitating cellular exchange of proteins, DNA, and RNA.^26^ GBM-derived EVs specifically contain miRNAs, mRNAs, rRNAs, tRNAs, and gene-regulating proteins.^27,28^ We have recently demonstrated that GBM EVs potently inducer immunosuppressive monocytes in a partly PD-L1-dependent fashion. These monocytes inhibit T cell proliferation.^29^ Others have also reported that GBM EVs can directly inhibit T cell responses in a PD-L1-dependent manner.^30^ Regardless, it is clear that EVs are important for intercellular communication with implications for tumor progression, angiogenesis, and immune tolerance.^31^

Based on this, we hypothesized that IFN-γ exposure causes GBM cells to release EVs with superinduced immunosuppressive capacity reflecting PD-L1 and IDO1 upregulation. Herein, we present evidence testing this hypothesis with human GBM cells, EVs, monocytes, and T cells *in vitro*.

## Materials and Methods

### Cell Culture

Human GBM cell lines dBT114, dBT116, dBT120, and dBT165 have been previously established in our laboratory from operative specimens as previously described.^29^ Lines were derived from Mayo Clinic in Rochester, MN (Mayo Clinic IRB312-003458). They were originally derived as stem-like lines in serum-free media but are now carried in culture as differentiated GBM lines at 37°C in 5% CO_2_ in DMEM/F12 (Gibco) with 10% fetal bovine serum (FBS). These remain neoplastic but, unlike the stem-like parent lines, grow as a monolayer not as spheres and express mature glioneuronal markers. Cells (2.5 × 10^5^) were seeded in 6-well plates. After 24 h, media was replaced with serum-free DMEM/F12 and treated with IFN-γ (100 ng/mL) or control.

### Isolating EVs by Differential Ultracentrifugation

Differential ultracentrifugation and nanoparticle tracking analysis (NanoSight) were our primary means to isolate and identify EVs from culture media, as previously described.^32^ Briefly, GBM-conditioned media was centrifuged at 1200 rpm for 3 min to eliminate cells and debris. The supernatant was then centrifuged at 3000 rpm for 10 min followed by ultracentrifugation in a Beckman L-60 ultracentrifuge (Beckman) at 24 000 rpm for 16 h. Supernatant was aspirated and EVs were resuspended in serum-free media. Nanoparticle tracking analysis (NanoSight NS300 Nanoparticle Characterization System; Malvern Panalytical) was performed to determine EV frequency and size.

### Isolating EVs by Density Gradient Ultracentrifugation

Density gradient EV isolation was performed as we have previously described.^33^ Briefly, GBM-conditioned media was collected after 72-h incubation and centrifuged at 1200 rpm for 5 min twice to remove remaining cells and debris. Supernatant (15 mL) was transferred to an Amicon Ultra-15 10,000 NMWL device (UFC901008) and further concentrated to 1 mL by centrifugation at 4000*g* for 20 min at 4°C. The OptiPrep diluent was prepared using 10% sucrose, 6 mM EDTA, 120 mM Tricine at pH 7.8 in water. Buffer A was prepared using 100 mL of 2.5 M sucrose, 1 mM EDTA, 20 mM Tricine at pH 7.8 in water. 45 mL of OptiPrep density gradient medium (Sigma-Aldrich, D1556) and 9 mL of OptiPrep diluent were mixed to yield a 50% OptiPrep solAn initial 2.9 mL layer of 40% of OptiPrep solution (3.2 mL of 50% OptiPrep solution plus 0.8 mL of Buffer A) was placed at the bottom of an ultra-clear centrifuge tube (Beckman Coulter No. 344060), followed by 2.9 mL of 20% OptiPrep solution, 2.9 mL of 10% OptiPrep solution, and 2.5 mL of 5% OptiPrep solution. Lastly, a 0.7-mL layer of concentrated cell culture media was placed on the top of the gradient solutions. This gradient underwent ultacentrifugation at 100 000*g* for 18 hat 4°C (Beckman) with maximum acceleration and minimum deceleration. After ultracentrifugation, 1 mL fractions were collected from top to bottom (12 fractions total for each ultracentrifugation gradient). EV concentration size and frequency in each fraction were determined by nanoparticle tracking analysis (NanoSight; Malvern, NanoSight NS300). EV protein concentration was quantified using the Pierce BCA protein Assay (REF 23228, Thermo Scientific) following the manufacturer’s instructions. The fraction with the highest EV concentration and lowest free protein concentration was used for subsequent experiments. Interfering RNA (RNAi)-mediated PD-L1 and IDO1 knockdown.

Short interfering RNA (siRNA) sequences targeting PD-L1 (sc-39699), IDO1 (sc-45939), or a nontargeting siRNA control (sc-37007) were acquired (Santa Cruz Inc). Each siRNA product consisted of pools of 3–5 target-specific 19–25 nt siRNAs designed to knock down expression of the gene of interest. GBM cells (2.5 × 10^5^) were incubated in DMEM/F12 with 10% FBS and siRNAs were transfected using Lipofectamine 2000 reagent (Invitrogen) per the manufacturer’s instructions. Cells were then recovered in complete medium for 24 h, and the effect on gene targeting was assessed by western blotting.

### Western Blotting

Whole-cell lysates (WCL) and EV samples were prepared with RIPA buffer (50 mM Tris [pH 7.4], 1% Triton X-100, 0.25% sodium deoxycholate, 150 mM NaCl, 1 mM EDTA [pH 8], and 10 mM NaF) containing cOmplete Protease Inhibitor (Roche). Proteins were then separated by electrophoresis on 4%–20% sodium dodecyl sulfate–polyacrylamide gel electrophoresis. Following membrane transfer, the proteins were probed using the following antibodies: (PD-L1 [#13684S], IDO1 [#86630S], CD9 [#13174S], calreticulin [#12238S], CD81 [#56039S], HSP90 [#4874S] (Cell Signaling), and CD63 [sc-5275] (Santa Cruz Inc)). Secondary antibody was horseradish peroxidase-conjugated goat antirabbit or goat antimouse (Jackson ImmunoResearch). Detection was by enhanced chemiluminescence.

### Monocyte and T Cell Isolation

Discarded, anonymized, healthy donor leukoreduction chambers were obtained from our institutional blood bank. Peripheral blood mononuclear cells (PBMCs) were separated from whole blood via Ficoll gradient centrifugation at 800*g* for 15 min. Isolated PBMCs were then resuspended in MACS buffer (phosphate-buffered saline, 2% bovine serum, 0.4% EDTA) and centrifuged at 800*g* for 10 min. Monocytes and T cells were isolated via CD14^+^ and CD3^+^ magnetic beads (Miltenyi Biotec), respectively, per the manufacturer’s instructions.

### Myeloid-Derived Suppressor Cell and Nonclassical Monocyte Induction

CD14^+^ monocytes (1 × 10^5^ cells) in serum-free media were seeded in 96-well plates in the presence or absence of 20 µg/well of GBM cell-derived EVs. These were then incubated for 72 h at 37°C under hypoxic (1% O_2_) conditions. Monocytes were then harvested and resuspended in serum-free media.

### Flow Cytometry

To quantify induction of myeloid-derived suppressor cells (MDSCs) and nonclassical monocyte (NCM), monocytes were resuspended in MACS buffer and stained with the following markers: CD11b:PE-Cy7, HLA-DR:BV421, CD16:BV785, PD1:PE (BioLegend, #101216, #307636, #302046, #329906), CD14:FITC, CD3-PerCP (Invitrogen, 11-0149-42, 67-0036-T100). To compare PD-L1 levels following IFN-γ stimulation, monocytes were incubated with EVs derived from GBM cells that had been incubated +/−IFN-γ and were stained with PD-L1:Alexa 596 (BD Pharmingen). In either case, cells were incubated with the antibodies at room temperature for 30 min. After washing with MACS buffer, cells were then fixed in 2% paraformaldehyde. All flow cytometry experiments were carried out using the LSRII flow cytometer with data analyzed by FlowJo (BD Life Sciences).

### T Cell Proliferation Assay

CFSE-stained T cells were seeded on anti-CD3 coated 96-well plates or stimulated T cells were plated at 1 × 10^5^ cells per well with αCD3/αCD28 DynaBeads (Thermo Fisher). Monocytes previously conditioned with EVs derived from untreated or IFN-γ-stimulated GBM cells were resuspended in serum-free DMEM/F12 and added to T cells in a 3:1 monocyte:T cell ratio. After 5 days of incubation, T cells were harvested and analyzed by flow cytometry as per above.

### Statistical Analysis

All data are presented as mean ± SEM. One- or 2-way ANOVA followed by Dunnett’s test (or Tukey’s test) was used to evaluate statistical significance using GraphPad Prism 8 software (GraphPad Software, Inc). Statistical significance was set at *P* < .05 and reported as *P < .05, **P < .01, ***P < .005, or ****P < .001.

## Results and Discussion

### IFN-γ Induces PD-L1 and IDO1 Expression in GBM Cells

**Figure 1.**
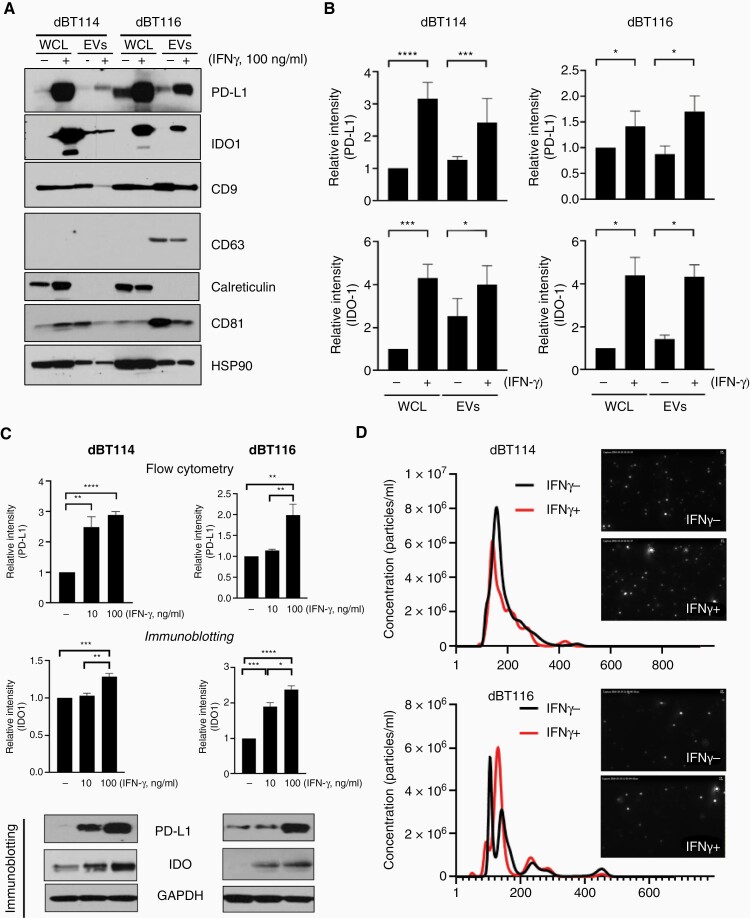
IFN-γ increases PD-L1 and IDO1 expression in human glioblastoma cells and extracellular vesicles. (A) Glioblastoma cell lines dBT114 or dBT116 ± 100 ng/mL IFN-γ for 24 h and expression of indicated proteins in whole-cell lysate (WCL) and extracellular vesicles (EVs) was assessed by western blot. (B) Immunoblots from (A) were normalized to loading control (HSP90) by densitometry. Relative intensity compared to baseline (WCL without IFN-γ exposure) is shown (mean ± SEM; *n* = 3). (C) dBT114 or dBT116 cells ± 10 or 100 ng/mL IFN-γ for 24 h and analyzed by western blot for IDO1, PD-L1, and GAPDH. Bar graphs for PD-L1 show median fluorescence intensity on flow cytometry relative to EVs without IFN-γ exposure (median ± standard deviation, *n* = 3) while those for IDO1 show relative densitometry intensity compared to baseline (no IFN-γ) after normalization to GAPDH (mean ± standard deviation; *n* = 3). (D) Nanoparticle tracker analysis histograms and photomicrographs showing the size distribution and frequency of dBT114 and dBT116 EVs ± 100 ng/mL IFN-γ. *P < .05, **P < 0.01,***P < .001, ****P < .0001. IDO1, indoleamine 2,3-dioxygenase 1; IFN-γ, interferon-gamma; PD-L1, programmed cell death ligand 1.

IFN-γ or control was added to dBT114 and dBT116 differentiated GBM cells after which WCL and EVs were isolated by differential ultracentrifugation. Of note, these EVs expressed EV-associated proteins (CD9, CD63, CD81, and HSP90) to varying degrees but did not express cytosolic proteins such as calreticulin (Figure 1A). PD-L1 and IDO1 expression were increased in both WCL and EVs of cells treated with IFN-γ (Figure 1A). On spectrophotometric densitometry, PD-L1 and IDO1 expression was significantly higher for each cell line after normalization to HSP90 (Figure 1B). IFN-γ-mediated induction of PD-L1 was dose dependent as determined by flow cytometry (confirming increased surface expression) and immunoblotting (Figure 1C and Supplementary Figure S1). Optimal IFN-γ concentration for maximal PD-L1 and IDO1 induction was 100 ng/mL and was used for all subsequent experiments. Nanoparticle tracking analysis confirmed the presence of EVs measuring between 50 and 300 nm diameter (Figure 1D). Prior published data from our laboratory have demonstrated that EVs isolated by differential ultracentrifugation from these cells lines appear similar when visualized by fluorescent microscopy and nanoscale flow cytometry.^29^ Electron microscopy demonstrates similar results (data not shown). IFN-γ treatment did not change the size or concentration of EVs released from the dBT114 and dBT116 GBM cell lines as characterized by nanoparticle tracking analysis (Figure 1D) nor were there any significant changes in expression of the EV markers CD63, CD9, and CD81 (Supplementary Figure S1).

These findings are consistent with published data in which PD-L1 expression was upregulated by IFN-γ in glioma stem cells with the increased expression found to anatomically correlate with the known IFN-γ response gene, IFN-γ response factor 1.^30^ However, we chose to focus on differentiated GBM cells rather than glioma stem cells given our prior findings demonstrating that differentiated GBM-derived EVs induce more immunosuppressive changes and have higher baseline PD-L1 expression than stem-like GBM-derived EVs.^29^ We also found increased expression of IDO1 in response to IFN-γ similar to prior reports in melanoma and hepatocellular carcinoma.^23,24^ IFN-γ is therefore capable of upregulating both PD-L1 and IDO1 in a dose-dependent manner in differentiated GBM cells and their EVs.

### IFN-γ-Treated GBM-Derived EVs Superinduce MDSCs and NCMs Without Directly Impacting T Cell Proliferation

GBM-derived EVs are thought to mediate immunosuppression although the precise mechanisms and clinical ramifications continue to be elucidated.^30,34^ We and others have reported GBM-derived EVs inhibit T cell proliferation indirectly via induction of MDSCs and NCMs.^29,34,35^ However, a recent study reported direct PD-LI-mediated inhibition of T cell activity and proliferation by GBM stem cell-derived EVs.^30^ We therefore sought to determine whether either INF-γ-treated or untreated GBM-derived EVs inhibit T cell proliferation directly. In keeping with our earlier study with untreated GBM EVs alone,^29^ there was no statistically significant difference in T cell proliferation when EVs derived from INF-γ-treated GBM cells versus EVs derived from untreated cells were incubated directly with isolated normal donor T cells despite the induction of PD-L1 and IDO1 in the former (data not shown). This does not eliminate the possibility of direct T cell effects beyond proliferation or in all circumstances as our focus on more differentiated GBM cell-derived EVs is distinct and our experimental methods may not be identical.

**Figure 2.**
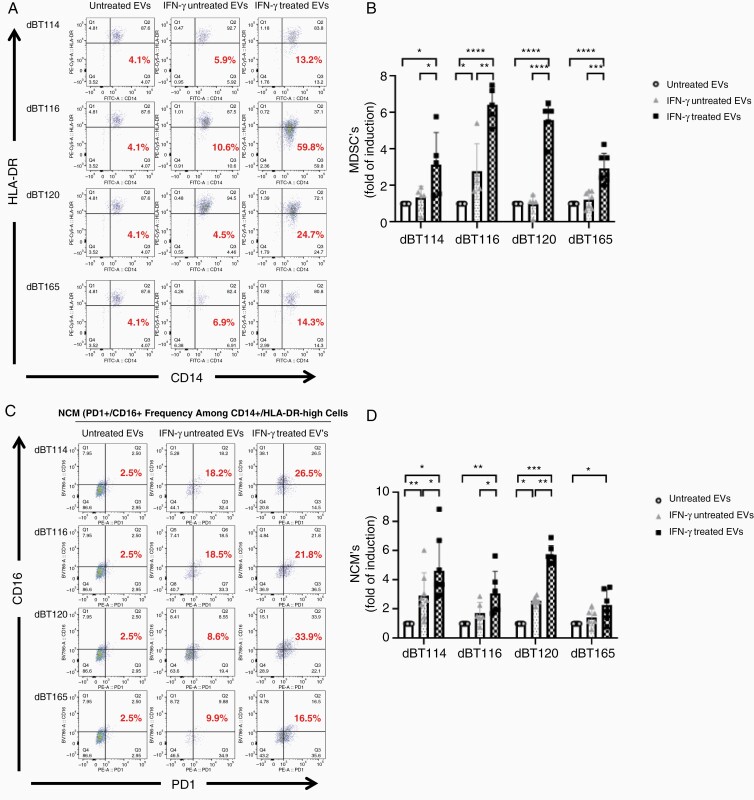
EVs from IFN-γ-treated glioblastoma cells cause superinduction of myeloid-derived suppressor cell (MDSC) and nonclassical monocyte (NCM) formation. Representative dot plots showing MDSC frequency (CD14^+^/HLA-DR^−^) (A) and NCM frequency (CD14^+^/PD-1^+^/CD16 +) (C) in monocytes in serum-free media, EVs, IFN-γ- (100 ng/mL) treated EVs (left lane, middle lane, right lane) from dBT cell lines. Bar graphs showing a significant induction in the mean frequency of CD14^+^/HLA-DR^−^ cells (B) and CD14^+^/PD1^+^/CD16^+^ cells (D). *P < .05, **P<0.01, ***P < .001, ****P < .0001. EVs, extracellular vesicles; IFN-γ, interferon-gamma.

Nevertheless, we found effects on monocytes more illuminating. GBM-derived EVs from the dBT114, dBT116, dBT120, and dBT165 lines without IFN-γ resulted in a mild (<2-fold compared to naive monocytes) MDSC increase for 1 cell line (dBT116) but not the other cell lines as determined by quantificating CD11b^+^/CD14^+^/HLD-DR^low^ cells on flow cytometry as previously described.^34,35^ However, following IFN-γ treatment, EVs from all 4 cell lines showed significant increases in MDSCs (>3-fold induction compared to baseline for EVs from all cell lines; Figure 2A and B). Similarly, EVs from IFN-γ-naive cell lines all showed a trend to NCM induction (CD14^+^/PD-1^+^/CD16^+^/HLA-DR^high^),^36–38^ but this was only significant for dBT114. In contrast, EVs derived from IFN-γ-exposed GBM cells induced significant increases NCM (>2-fold compared to baseline) for all cell lines (Figure 2C and D). MDSC and NCM induction by IFN-γ-treated GBM-derived EVs cells was reproducible with 4 different GBM cell lines and monocytes obtained from 4 distinct healthy donors (Figure 2).

Although our established method for isolating EVs from conditioned media by differential ultracentrifugation^28,29^ clearly yields particles of EV size and shape that express EV-associated proteins (CD9, CD63, CD81, and HSP90) and do not express cytosolic proteins like calreticulin (Figure 1A), we cannot rule out the presence of additional soluble proteins released into culture media. Published guidelines from the International Society for EVs consider differential ultracentrifugation to be an “intermediate specificity, intermediate yield” technique for EV isolation.^39^ Indeed, we have previously developed techniques using density gradient ultracentrifugation for plasma EV isolation to avoid this issue.^33,40^ Therefore, we compared MDSC and NCM induction by GBM-derived EVs purified from the same conditioned media by either differential ultracentrifugation or density gradient ultracentrifugation. This allowed isolation of EVs with high frequency but low soluble protein concentration from the mid-portion of our density gradient ultracentrifugation fractions (Supplementary Figure S2A). As with EVs isolated by differential ultracentrifugation, this yielded particles measuring between 50 and 400 nm by nanoparticle tracking analysis (data not shown) that expressed EV-associated proteins (CD9, CD63, CD81, and HSP90) but not cytosol-specific proteins (calreticulin). IDO1 and PD-L1 expression was upregulated in response to IFN-γ (Supplementary Figure S2B). Exposing normal monocytes to GBM EVs induced differentiation into MDSCs and NCMs which was further increased with exposure to IFN-γ-conditioned GBM EVs in a manner that was essentially identical regardless of whether these EVs were isolated by differential ultracentrifugation or density gradient ultracentrifugation, with the possible exception of slightly less robust NCM induction by density gradient ultracentrifugation-isolated EVs (Supplementary Figure S2C–F). Given these findings, subsequent experiments were limited to differential ultracentrifugation-isolated EVs for simplicity.

Our data show robust induction of the MDSC and NCM populations when normal human donor monocytes are incubated with EVs derived from GBM cells stimulated with IFN-γ. NCMs are associated with inhibition of T cell-mediated antitumor immunity in an IL-10-dependent mechanism in human colorectal cancer.^41^ However, NCMs have been implicated in divergent roles in cancer pathobiology including curtailing metastasis, neutrophil and natural killer cell recruitment and angiogenesis.^42^ The functional overlap of NCMs and MDSCs is considerable and MDSCs have also been implicated in cancer angiogenesis, drug resistance, promotion of tumor metastasis, and overall tumor immunosuppression.^43^ Mechanistic understanding of how these induced pathologic cell populations orchestrate complex protumor biological activities may provide critical insight for the development of potentially novel therapeutics.

### PD-L1 and IDO1 Are Required for IFN-γ-Mediated Induction of MDSCs and NCMs

**Figure 3.**
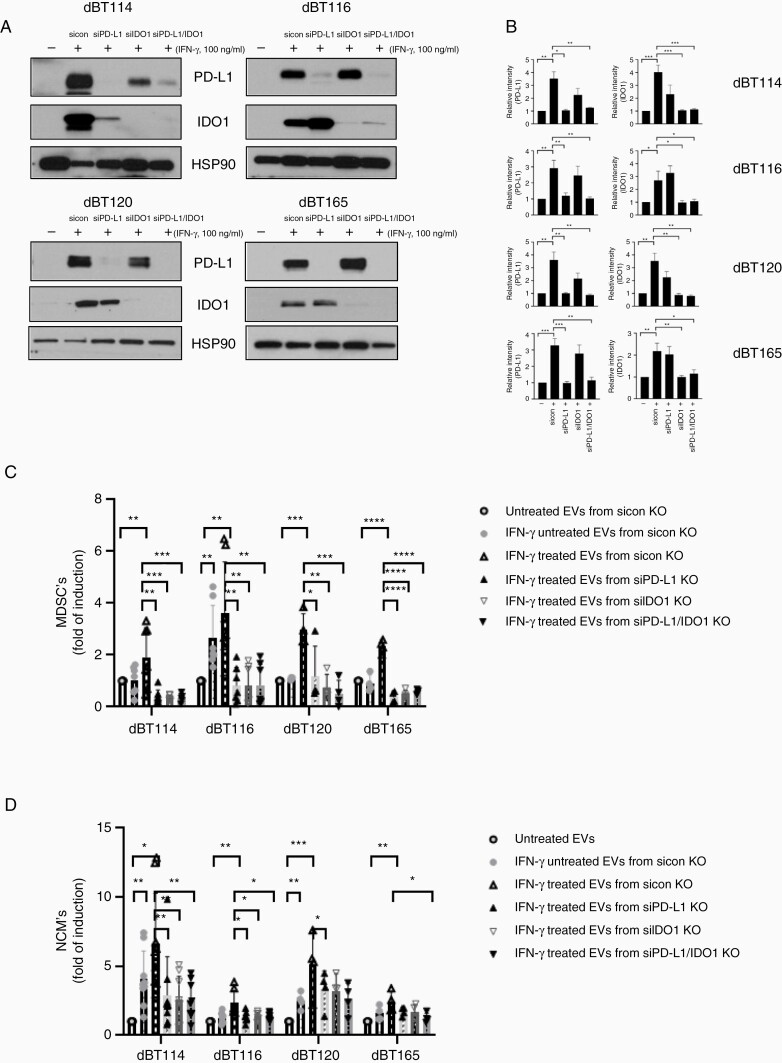
PD-L1 and IDO1 expression are both required for MDSC and NCM superinduction in response to IFN-γ-exposed GBM EVs. (A) dBT114, dBT116, dBT120, and dBT165 cells were transfected with either nontargeting (sicon), PD-L1 (80 nmol/L) or IDO1 (80 nmol/L). After 72 h, the transient transfectants were stimulated in the absence (−) or presence (+) of 100 ng/mL IFN-γ for 24 h and western blotted for PD-L1, IDO1, and HSP90 as a loading control. (B) Immunoblots from (A) were normalized to loading control (HSP90) and evaluated. Bar graphs showing mean frequency of MDSC (C) or NCM (D) induction in serum-free media, EVs, EVs from dBT cell lines, PD-L1 knockdown dBT cells or IDO1 knockdown dBT cells treated with (+) or without (−) IFN-γ (100 ng/mL) for 3 days. Note that both PD-L1 and IDO1 knockdown markedly reduced the superinduction of MDSC and NCM in response to IFN-γ. No synergy or additive effects are seen. *P < .05, **P<0.01, ***P < .001, ****P < .0001. EVs, extracellular vesicles; GBM, glioblastoma; IDO1, indoleamine 2,3-dioxygenase 1; IFN-γ, interferon-gamma; MDSC, myeloid-derived suppressor cell; NCM, nonclassical monocyte; PD-L1, programmed cell death ligand 1.

To determine whether PD-L1 and IDO1 are *necessary* for EV-mediated MDSC and NCM induction following IFN-γ treatment of the GBM cells, an RNAi strategy was employed. siRNA directed against PD-L1 led to a significant reduction of PD-L1 signal in WCL and EVs compared to siRNA control in the presence of IFN-γ but did not affect IDO1 levels. Similarly, siIDO1 resulted in significant reduction of IDO1 levels in both WCL (Supplementary Figure S3) and EVs (Figure 3). Expression levels of PD-L1 and IDO1 were simultaneously reduced in the presence of both siPD-L1 and siIDO1 to levels reminiscent of that seen with exposure to either RNAi alone. Expression levels were unaltered by a nonspecific RNAi control (Figure 3A and B and Supplementary Figure S3).

Superinduction of MDSCs by EVs derived from IFN-γ-treated GBM cells was reduced to levels below untreated monocytes when GBM cells were also exposed to either siPD-L1 or siIDO1 alone and in the presence of both siIDO and siPD-L1. This reduction was statistically significant (Figure 3C). Similarly, siIDO and siPD-L1 incubated with GBM cells alone or in combination, significantly reduced the IFN-γ-treated EV-mediated increase in NCMs (Figure 3D).

Our data demonstrate that IFN-γ increases both PD-L1 and IDO1 expression in GBM EVs. These EVs induce normal human monocytes to differentiate into MDSCs and NCMs and this is dependent on both PD-L1 and IDO1. Coexpression of PD-L1 and IDO1 have been reported in squamous cell carcinoma,^44^ lung cancer,^45^ and melanoma.^46^ In GBM, IDO1 and PD-L1 are expressed in both tumor and nonneoplastic cells although 1 report suggests that it is the nonneoplastic IDO1 that contributes to PD-L1-mediated T cell dysfunction and immunosuppression in a mouse GBM model.^47^

### Monocytes Treated With IFN-γ-Treated GBM-Derived EVs Inhibit T Cell Proliferation in a PD-L1- and IDO1-Dependent Manner

**Figure 4.**
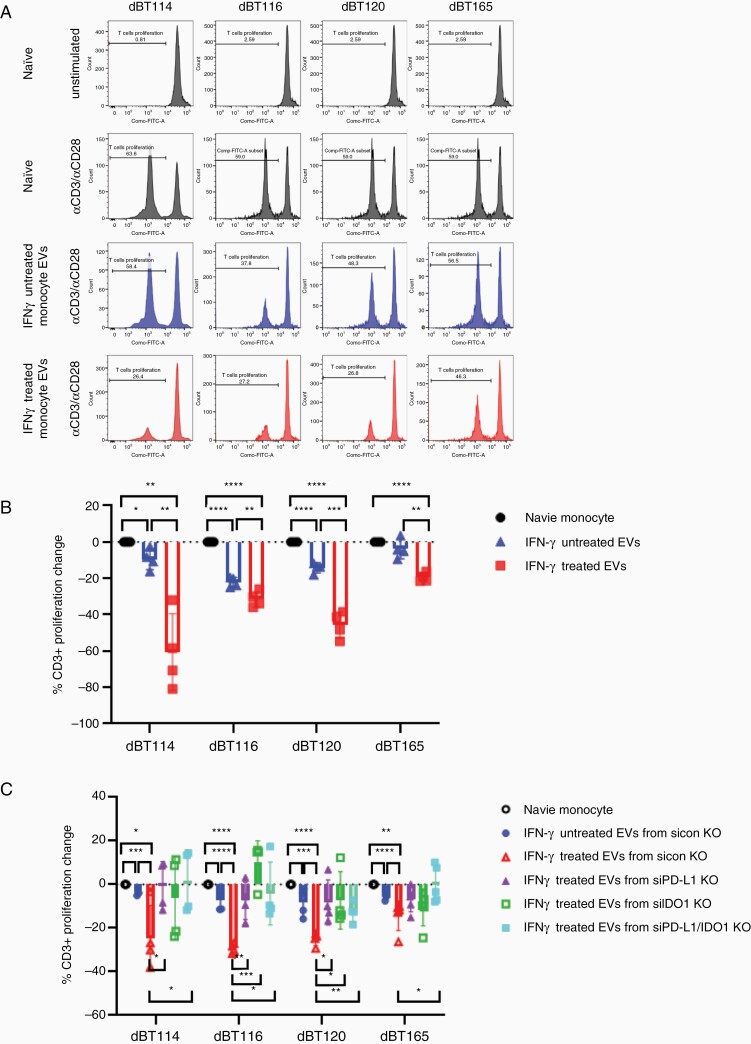
Superinduction of T cell inhibition by monocytes cultured with IFN-γ-exposed GBM EV depends on GBM PD-L1 and IDO1 expression. (A) Representative histograms showing proliferation of CFSE-stained T cells stimulated with or without anti-CD3/anti-CD28 in serum-free media alone, with naive GBM EV-treated monocytes, or with IFN-γ-treated GBM EVs. (B) Bar graphs showing mean T cell proliferation (from 4 donors) in response to anti-CD3/anti-CD28 antibodies in the conditions outlined. (C) Bar graphs showing mean T cell proliferation in response to anti-CD3/anti-CD28 antibodies in serum-free media alone or in the presence of monocytes exposed to GBM EVs, GBM EVs with PD-L1 knockdown, or GBM EVs with IDO1 knockdown. GBM cells treated with (+) or without (−) IFN-γ (100 ng/mL) for 3 days prior to EV harvest. *P < .05, **P<0.01, ***P < .001, ****P < .0001. EV, extracellular vesicle; GBM, glioblastoma; IDO1, indoleamine 2,3-dioxygenase 1; IFN-γ, interferon-gamma; PD-L1, programmed cell death ligand 1.

We have previously demonstrated that GBM-derived EVs indirectly inhibit T cell proliferation via induction of MDSCs and NCMs.^29^ To determine whether monocytes treated with EVs derived from IFN-γ-treated GBM cells further enhance the inhibitory effects on anti-CD3/anti-CD28-mediated T cell proliferation, monocytes from 4 normal human donors were incubated with EVs derived from 4 GBM cell lines +/−IFN-γ exposure. Monocytes cultured with EVs from untreated GBM cells led to significantly decreased T cell proliferation (Figure 4A and B). This reduction in T cell proliferation was even more robust when monocytes were cultured with IFN-γ-treated GBM-derived EVs (Figure 4A and B). RNAi-mediated knockdown of PD-L1 and IDO1 in IFN-γ-treated GBM-derived EVs restored anti-CD3/anti-CD28-mediated T cell proliferation to baseline when exposed to monocytes preincubated with GBM EVs (Figure 4C). It is subject to debate whether anti-CD3/anti-CD28 stimulation or anti-CD3 stimulation alone is more appropriate for assessing the impact of costimulatory molecules or their homologs like PD-L1 on T cell proliferation.^29,30^ Therefore, we repeated our T cell proliferation experiments with anti-CD3 stimulation alone. Similar data were seen in this system when T cell stimulation was limited to anti-CD3 (Supplementary Figure S4).

The evidence supporting IFN-γ-mediated upregulation of IDO1 and PD-L1 in a variety of cancers is strong.^24,44–49^ Once upregulated, IDO1 and PD-L1 facilitate tumor-mediated immunosuppression through a number of proposed mechanisms which converge on T cell-mediated immunological responses. This is thought to occur primarily by either inhibiting T cell function or decreasing T cell numbers (through apoptosis and/or decreased proliferation). IDO1 and PD-L1 levels are increased in GBM EVs following IFN-γ exposure. We have not demonstrated any direct T cell inhibition by GBM EVs even with upregulated IDO1 and PD-L1 expression. In keeping with our prior studies,^29^ we demonstrate indirect T cell inhibition by MDSCs and NCMs induced from normal monocytes in response to GBM EVs in a manner dependent on PD-L1 and IDO1. Indeed, systemic T cell levels are reduced in GBM patients with profound effects on systemic and local immune function.^20,50^

In summary, we demonstrate that IFN-γ upregulates PD-L1 and IDO1 in differentiated GBM cells and their EVs. We show that PD-L1 and IDO1 in EVs induce MDSCs and NCMs in normal monocytes which in turn inhibit T cell proliferation. This paradoxical response to a proinflammatory cytokine (IFN-γ) commonly released during immune responses is a major potential barrier to successful immunotherapy. These findings suggest new therapeutic avenues by targeting GBM EV-mediated immunosuppression. The data also suggest that a combinatorial strategy targeting both PD-L1/PD-1 interactions along with IDO1 may provide synergism in GBM immunotherapeutics.

## Supplementary Material

vdac017_suppl_Supplementary_Figure_S1

vdac017_suppl_Supplementary_Figure_S2

vdac017_suppl_Supplementary_Figure_S3

vdac017_suppl_Supplementary_Figure_S4
