# Supplementary figures and images for: Superinduction of immunosuppressive glioblastoma extracellular vesicles by IFN-γ through PD-L1 and IDO1
Source: Neurooncol Adv. 2022 Feb 15;4(1):vdac017. doi: 10.1093/noajnl/vdac017 (PMC9389426; doi:10.1093/noajnl/vdac017)

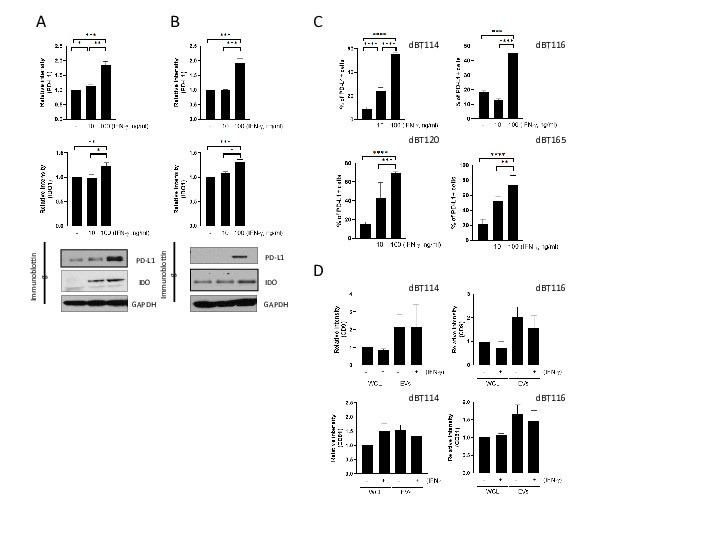

Supplement: vdac017_suppl_Supplementary_Figure_S1 [file vdac017_suppl_supplementary_figure_s1.jpeg]

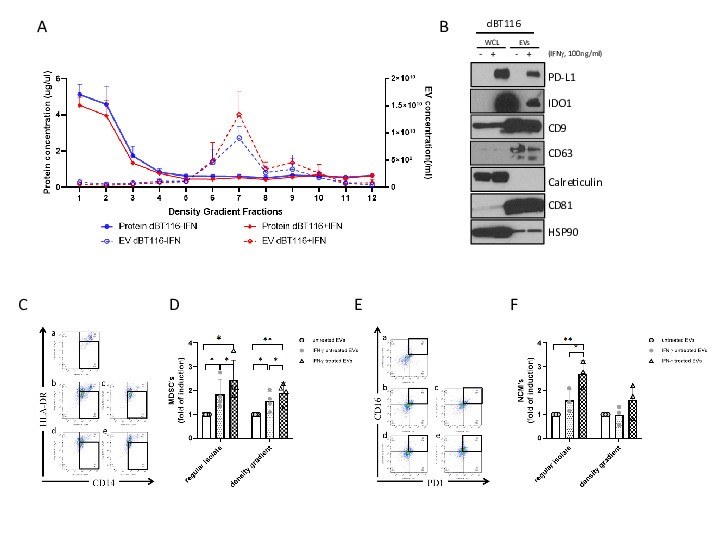

Supplement: vdac017_suppl_Supplementary_Figure_S2 [file vdac017_suppl_supplementary_figure_s2.jpeg]

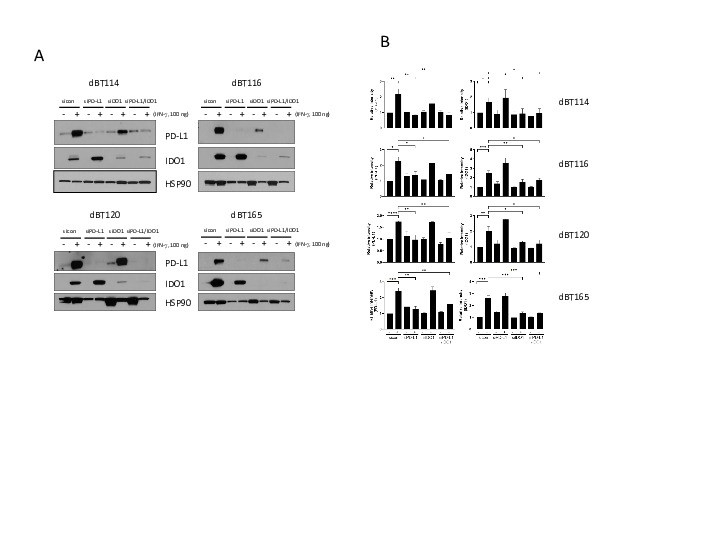

Supplement: vdac017_suppl_Supplementary_Figure_S3 [file vdac017_suppl_supplementary_figure_s3.jpeg]

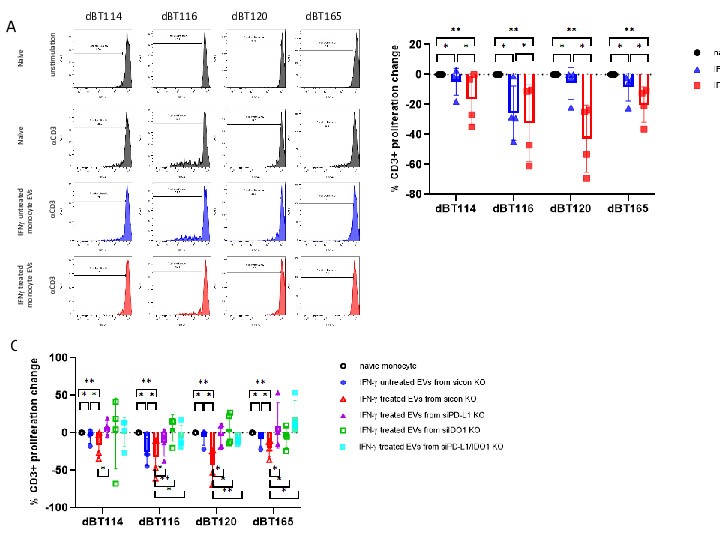

Supplement: vdac017_suppl_Supplementary_Figure_S4 [file vdac017_suppl_supplementary_figure_s4.jpeg]
